# A pilot cohort analytic study of Family Integrated Care in a Canadian neonatal intensive care unit

**DOI:** 10.1186/1471-2393-13-S1-S12

**Published:** 2013-01-31

**Authors:** Karel O’Brien, Marianne Bracht, Kristy Macdonell, Tammy McBride, Kate Robson, Lori O’Leary, Kristen Christie, Mary Galarza, Tenzin Dicky, Adik Levin, Shoo K Lee

**Affiliations:** 1 Department of Paediatrics, University of Toronto, Toronto, M5G 1X5, Canada; 2 Neonatal Intensive Care Unit, Mount Sinai Hospital, Toronto, M5G 1X5, Canada; 3 Formerly at Newborn and Premature Children’s Department, Tallinn Children’s Hospital, Tallinn, Estonia; 4 Departments of Paediatrics, Obstetrics & Gynaecology, and Public Health, University of Toronto, Toronto, M5G 1X5, Canada

## Abstract

**Background:**

We have developed a Family Integrated Care (FIC) model for use in a neonatal intensive care unit (NICU) where parents provide most of the care for their infant, while nurses teach and counsel parents. The objective of this pilot prospective cohort analytic study was to explore the feasibility, safety, and potential outcomes of implementing this model in a Canadian NICU.

**Methods:**

Infants born ≤35 weeks gestation, receiving continuous positive airway pressure or less respiratory support, with a primary caregiver willing and able to spend ≥8 hours a day with their infant were eligible. Families attended daily education sessions and were mentored at the bedside by nurses. The primary outcome was weight gain, as measured by change in z-score for weight 21 days after enrolment. For each enrolled infant, we identified two matched controls from the previous year’s clinical database. Differences in weight gain between the two groups were analyzed using a linear mixed effects multivariable regression model. We also measured parental stress levels using the Parental Stress Survey: NICU, and interviewed parents and nurses regarding their experiences with FIC.

**Results:**

This study included 42 mothers and their infants. Of the enrolled infants, matched control data were available for 31 who completed the study. The rate of change in weight gain was significantly higher in FIC infants compared with control infants (p < 0.05). There was also a significant increase in the incidence of breastfeeding at discharge (82.1 vs. 45.5%, p < 0.05). The mean Parental Stress Survey: NICU score for FIC mothers was 3.06 ± 0.12 at enrolment, which decreased significantly to 2.30 ± 0.13 at discharge (p < 0.05). Feedback from the parents and nurses indicated that FIC was feasible and appropriately implemented.

**Conclusions:**

This study suggests that the FIC model is feasible and safe in a Canadian healthcare setting and results in improved weight gain among preterm infants. In addition, this innovation has the potential to improve other short and long-term infant and family outcomes. A multi-centre randomized controlled trial is needed to further evaluate the efficacy of FIC in the Canadian context.

## Background

In the highly technological environment of the modern neonatal intensive care unit (NICU), infants are physically, psychologically, and emotionally separated from their parents. To address this issue, many programs, such as kangaroo care, skin-to-skin care, and family-centred care, encourage greater parent involvement [1-3]. However, most programs still adhere to the common premise that only NICU professionals with special skills can provide care for the infant, and parents are generally relegated to a supportive role. Some parents have described themselves as “voyeurs” who are “allowed” to visit and hold their infants. Many feel anxious and unprepared to care for their infants after discharge [4].

A review of family-centred care in NICUs by Gooding *et al.*, in 2011 [5], noted that many models and programs have been developed to promote family-centred care in North America, including the efforts of the Institute for Patient and Family Centered Care, the American Academy of Paediatrics, the Vermont Oxford Collaborative, the Family Centered Care map, the March of Dimes, and the Creating Opportunities for Parent Empowerment (COPE) program. Many of the programs, such as COPE, have focused on a specific aspect of family-centred care, such as a parental education and behavioural interventions, and have shown benefits to infants and parents [6]. However, the review concluded that despite all these efforts there has been poor dissemination of family-centred care practices into NICUs and few large randomised controlled studies of most family-centred models of care. In another review of interventions for preterm infants included in this supplement, Benzies *et al.*, suggest that providing psychosocial supports for parents may have the greatest long term benefits for families [7].

Several reports from outside North America have also suggested that parents can play a larger role in providing direct care of infants in the NICU and that there may be many short and long-term benefits of this practice. However, these practices have not previously been adopted in North America [1-3,8]. In our Family Integrated Care (FIC) model, which was implemented in this pilot study, we adopted a major paradigm shift by including parents as an integral part of the NICU team so that they could provide active care for their infant, instead of being in a passive support role. During their participation in the FIC program, parents learned how to provide all care (except for intravenous fluid administration and medications) for their infants in the NICU, while the nurses became educators and coaches for the parents.

FIC is a program designed by a multidisciplinary steering committee that included parents of prior NICU infants (‘veteran parents’), a physician, nurses, a parent educator, a lactation consultant, and a social worker. The program was based on the ‘Humane Neonatal Care’ model developed by Adik Levin in Tallinn, Estonia [2]. Education of the parents and the nurses to support their role changes was seen as a key component of the program and two curricula were developed for this as part of the program. In addition to education, we also provided additional physical and psychological supports for the FIC parents. Physical supports included parking/transit passes, rest/sleep room, a kitchen area, and screens and breast pumps at the bedsides. Psychological support was provided by veteran parents who had prior experience of having an infant admitted to the NICU. One of three veteran parents was available in the NICU for a half-day a week, where they led or co-led the FIC education session scheduled for that day. Each veteran parent also facilitated a recreational activity, such as arts and crafts or a coffee hour, in order to develop a sense of community within the FIC program, and was available to FIC parents by phone or email. Support for the nurses included the availability of two nurses who were members of the FIC steering committee and a study coordinator to help facilitate and answer caregiver questions at the bedside. Increased social work support was also available to address any problems that arose, such as issues with communication between parents, veteran parents, and healthcare providers.

The purpose of the prospective matched case-control pilot study described here was to: 1) Explore the feasibility and safety of the FIC model in a Canadian NICU, and 2) Identify potential improvements in neonatal and parental outcomes.

## Methods

### Study design

This pilot study of FIC was a cohort analytic study, in that the cohorts were identified before implementation of the intervention (FIC) rather than based on outcome, as is the case in a case-control study. The cohort analytic design is superior to a case-control study as it provides stronger evidence of causality [9].

### Study recruitment and ethics

Infants admitted to the NICU between 1^st^ March, 2011 and 30^th^ April, 2012 were eligible to participate in the study when they became stable on continuous positive airway pressure (CPAP) or required less respiratory support, and were receiving greater than 50% of their fluid requirements as enteral feeds. As this was a pilot study, we enrolled only 4 patients at a time on a rolling basis. Following admission to the NICU, as infants became eligible their parents were provided with an information package about the FIC study and approached to consent to, and complete a Parental Stress Scale: Neonatal Intensive Care Unit (PSS-NICU) questionnaire. When a bed space was available, and if there were no immediate plans for transfer, we approached the family for written consent to participate in FIC. The parent who committed to be the primary caregiver signed the consent form and completed a demographic questionnaire. The partners were invited to participate to the best of their ability in the FIC program but specific demographic data were not collected from them.

Infants were excluded if they had severe congenital anomalies, were receiving palliative care, were critically ill and deemed unlikely to survive, were scheduled for early transfer, or their parents were unable to participate due to health, social, or language issues that would inhibit their communication with the medical and nursing team. The study was approved by our institutional Research Ethics Board.

### Family integrated care protocol

Parents enrolled in the FIC program were oriented to the program, the supports, the tools provided for their self education, and the charting required by a research nurse. As soon as enrolled, the primary caregiver was asked to commit to spending ≥8 hours per day in the NICU between 7 am and 8 pm, including attendance at daily medical rounds and a daily education session Monday to Friday for at least 3 weeks or until discharge. The daily education sessions were provided to all parents according to a set curriculum, were coordinated and led by a Parent Resource Nurse and/or a veteran parent, and were held at the bedside or in a classroom. Some sessions included material taught by other members of the multidisciplinary NICU team, such as a pharmacist, dietician, or lactation consultant. Additional one-to-one education was provided on an “as needed” basis. Parents were also expected to provide care for their infant(s), especially in the areas of feeding, bathing, dressing, holding, and providing skin-to-skin care, perform basic charting, and maintain a record of their own learning regarding their proficiency in providing care for their infant(s) in the NICU. Nurses remained responsible for more technical aspects of the infant’s care, such as insertion of nasogastric catheters, placement of CPAP prongs, oral suctioning, and adjustment of oxygen concentrations. Forty nurses who volunteered to participate in the project were provided with additional education on how to deliver the FIC program. These nurses were preferentially assigned to take care of FIC families as staffing permitted and provided one-on-one and small group education and coaching to the FIC parents.

### Infant measures

Demographic and treatment characteristics were collected from the infants enrolled in the study, including gestational age, birth weight, small for gestational age, singleton status, Apgar score at 1 min and 5 min, score of neonatal acute physiology version II (SNAPII), days on oxygen, days on CPAP, days on ventilation, surfactant administration, and caffeine administration. Control infants for infant-related outcomes were identified from the previous year’s NICU clinical database (January to December, 2010) and matched for gender, gestational age (± 2 weeks), birth weight (± 300 grams), age at enrolment, and length of stay following enrolment of ≥21 days. All factors were given equal weight and the controls were selected from the first match made for each enrolled infant. The same variables in the control group were extracted from the NICU clinical database.

The primary outcome of the study, which was selected *a priori*, was change in weight at 21 days following enrolment in the FIC program as measured by the z-score [10]. The z-score refers to the exact number of standard deviations greater or smaller than the median, and is used to monitor the growth of an infant relative to the expected intrauterine growth rate [10]. The score is standardized to population growth standards and more appropriate than percentiles for infants whose size lies outside the normal range of a growth chart [10]. Secondary infant outcomes included: (1) weight gain velocity at 21 days from enrolment in the FIC program; (2) rate of breastfeeding at enrolment and at discharge; (3) number of critical incident reports per 1000 patient days; (4) neonatal mortality; and (5) rates of the following major morbidities: (a) nosocomial infection, defined using the Centers for Disease Control criteria [11]; (b) necrotizing enterocolitis, defined using Bell’s criteria [12]; (c) bronchopulmonary dysplasia, defined according to Shennan *et al.*,[13]; (d) intraventricular hemorrhage, as classified by the Canadian Paediatric Society [14] from cranial ultrasound performed during the first 28 days of life; and (e) retinopathy of prematurity (ROP), staged according to the International Classification of Retinopathy of Prematurity [15].

### Parental measures

Demographic data were collected from the FIC parent in the caregiving role including their age, relationship status, number of other children, income, education level, distance travelled to the NICU, and ethnic background. We also measured the level of parental stress by asking the parent in the caregiving role to complete a PSS-NICU questionnaire [16] in the first week following admission and when their infant reached 35 weeks corrected age if still present in the unit. The PSS-NICU is a 34-item self report instrument used to measure parents’ perceptions of stress within the NICU [16,17]. Within the PSS-NICU questionnaire are 3 subscales that measure stress related to: (1) the sights and sounds of the unit (6 items), (2) the appearance and behaviour of the infant (17 items), and (3) the impact of the parents’ role and their relationship with their baby (11 items). Each subscale consists of a series of examples of potentially stressful aspects of the NICU environment, e.g., the presence of monitors and equipment, and parents were asked to rate their level of stress related to each item. The responses were scored on a 5-point Likert scale with ‘1’ being ‘Not at all stressful’ and ‘5’ being ‘Extremely stressful’ [16,17]. The internal consistency of the PSS-NICU has been previously demonstrated, with Cronbach alpha coefficients ranging from 0.73 to 0.96 for the subscales and from 0.89 to 0.94 for the total scale [16,18,19]. To provide a concurrent control group for measures of parental stress in the FIC parents, we also approached all parents of infants born at <35 weeks admitted to the NICU during the study period to complete the PSS-NICU. These parents were not enrolled in the FIC study simply due to the limited availability of FIC bed spaces.

### FIC program evaluation

A qualitative evaluation of the implementation, feasibility and acceptability of the FIC program, as well as parents’ and nurses’ perceptions of the benefits of the program was conducted using semi-structured one-to-one interviews. All FIC parents were interviewed prior to discharge or transfer and at neurodevelopmental follow-up 4 months post-discharge by a research coordinator who knew the families. The interview included an open ended question: “Describe your experience of FIC”, as well as series of questions regarding specific elements of the program, such as the supports provided, and the quality and quantity of the educational information provided. A total of 19 out of the 40 nurses who participated in the FIC program were interviewed on a one-to-one basis by a research assistant who did not work in the NICU 6 months following implementation of the program. The nurse interviews also examined their experiences with the program and its implementation. All interviews were recorded, transcribed and imported into NVIVO software for thematic analysis.

### Statistical analysis

Analysis of the differences in clinical outcomes was performed on an intention-to-treat basis using SAS software v9.2 (SAS Institute, Cary, North Carolina). Student’s t-test was used to compare the mean change in z-score for weight between the FIC and control groups. For the multivariable analyses, we examined the weight change after 21 days of enrolment (Wt21-Wt1) by testing the interaction (FIC x Time) using mixed-effect models to account for the repeated measurement of weight and correlated data due to the matched controls. Student’s t-test, the χ^2^ test and ANOVA were used to compare the secondary outcomes for continuous variables, categorical variables, and repeated measures, respectively. A p value of < 0.05 indicated statistical significance.

## Results

### Study enrolment

A total of 56 families were approached regarding enrolment in the FIC pilot study. Of the families 42 (75%) agreed to take part, with the mother agreeing to be the primary caregiver in every case. Fathers/partners were not precluded from participation and in several cases were observed to provide the majority of care at the weekends. The total number of infants enrolled was 46 (there were 4 sets of twins) but of these 4 infants were then excluded, 3 because of unanticipated transfer within the first week of enrolment (1 of those was a set of twins) and 1 because the infant became medically unstable with pulmonary hypertension. We then identified 2 control infants for each of the remaining infants based on gender, gestational age (± 2 weeks), birth weight (± 300 grams), age at enrolment, and length of stay following enrolment of ≥21 days. We were unable to find matched controls for 11 FIC infants based on the criteria, so the final study population was 31 infants with 62 matched controls.

### Infant characteristics and outcomes

At enrolment the mean age after birth of the infants in the FIC group was 30.8 days (standard deviation, 20.7 days). There were no differences in the characteristics of infants who were involved in the FIC program and the infants in the control group (Table 1). The FIC infants for whom a matched control could not be found had a median gestational age of 27 weeks (range, 23–34 weeks), a median birth weight of 1130 g (range, 590–1130 g), a median age at enrolment of 22 days (range, 7–90 days), and a median corrected gestational age at enrolment of 35.1 weeks (range, 29.1–35.9). With the exception of corrected gestational age, these figures are similar to those of the FIC infants with matched controls (see Table 1), suggesting that the older corrected age of the unmatched infants at enrolment was the reason matched controls could not be found. These infants would have been outliers in comparison with the general population of NICU infants.

**Table 1:** Comparison of characteristics for infants in FIC and matched control group

	FIC infants (n = 31)	Matched controls (n = 62)	
Characteristics	n (%)	n (%)	p-value^1^
**Gestational age (wk), mean (SD)**	27.5 (2.4)	27.7 (2.6)	0.31
**Birth weight (g), mean (SD)**	1106 (419)	1061 (389)	0.11
**Age at enrolment (days), mean (SD)**	30.8 (20.7)	N/A	N/A
**Corrected gestational age at enrolment (weeks), mean (SD)**	31.9 (2.1)	N/A	N/A
**Small for gestational age**	3 (9.7)	9 (14.5)	0.45
**Singleton n (%)**	22 (71.0)	39 (62.9)	0.47
**Apgar score <7 at 5 min**	6 (19.4)	7 (11.3)	0.28
**Apgar score <7 at 1 min**	16 (51.6)	34 (54.8)	0.74
**SNAPII score >20**	6 (19.4)	13 (21.0)	0.85
**Days on oxygen, median (IQ range)**	36 (2–61)	19 (4–61)	0.79
**Days on CPAP, median (IQ range)**	41 (13–55)	41 (25–55)	0.44
**Days on ventilation, median (IQ range)**	2 (0–6)	5 (1–11)	0.31
**Surfactant administration**	23 (74.2)	48 (77.4)	0.71
**Caffeine administration**	29 (93.5)	55 (88.7)	0.34

Notes: Data are for total hospital stay. ^1^A conditional logistic regression method accounting for matched case-control data was used. The Mantel-Haeszel test was also applied for categorical data. The results from both methods were consistent. CPAP, continuous positive airway pressure; FIC, family integrated care; IQ, interquartile range; N/A, not applicable; SD, standard deviation; SNAPII, score of neonatal acute physiology version II.

There was a 24.5% increase in weight gain as measured by the z-score of the FIC group compared with matched case controls, but the results were not statistically significant (p = 0.26) (Table 2). However, the use of a linear mixed effects multivariable regression model, adjusted for Apgar score at 5 min, SNAPII, maternal age, and birth weight showed that the rate of change in weight gain was significantly higher for the FIC group (34.5) compared with the control group (32.2; interaction term between time and FIC group 2.3, p < 0.01).

**Table 2:** Comparison of neonatal outcomes

Outcomes	FIC infants (n = 31)	Matched controls (n = 62)	p-value^1^	Percent change
**Zwt21 – Zwt1, mean (SD)**	0.61 (0.44)	0.49 (0.41)	0.26	24.5
**(Wt21-Wt1)/(Wt1/1000)/21, mean (SD)**	21.59 (6.43)	20.32 (6.59)	0.48	6.25
**(Wt21-Wt1)/21, mean (SD)**	30.82 (9.08)	28.25 (9.09)	0.17	9.1
**Nosocomial infection, N (%)**	0 (0.0)	6 (9.7)	0.057	-100
**Incidents / 1000 person days (95%CI)**	0.84 (0, 1.96)	1.15 (0, 3.08)	0.78	-26.96
**BPD, N (%)**	11 (35.5)	24 (38.7)	0.54	-8.27
**ROP3, N (%)**	0 (0.0)	8 (14.3)	0.045*	-100
**IVH grade 3 or 4 or PVL, N (%)**	2 (6.7)	5 (8.3)	0.78	-16.87
**NEC, N (%)**	0 (0.0)	2 (3.2)	0.31	-100
**Breast feeds (> 90%), n (%)**	23 (74.2)	25 (40.3)	0.002*	45.7

Notes: Weight was measured at study enrolment and 21 days after enrolment; all other outcomes are for the period from enrolment to discharge. ^1^A conditional logistic regression method accounting for matched case-control data was used. The Mantel-Haeszel test was also applied for categorical data. The results from both methods were consistent. BPD, bronchopulmonary dysplasia; CI, confidence interval; FIC, family integrated care; IVH, intraventricular hemorrhage; NEC, necrotizing enterocolitis; PVL, periventricular leukomalacia; ROP3, retinopathy of prematurity, stage 3; SD, standard deviation; Wt1, weight on enrolment; Wt21, weight 21 days after enrolment; Zwt1, z-score for weight on enrolment; Zwt21, z-score for weight 21 days after enrolment.

The secondary outcomes of the study are also included in Table 2. There was a statistically significant decrease (p < 0.05) in the incidence of stage 3 or higher ROP (0 vs. 14.3%) between study enrolment and discharge, and an increase in the incidence of breastfeeding at discharge (82.1 vs. 45.5%). When compared with the control group, there was a decrease in the incidence of nosocomial infection (0 vs. 9.7%) and number of incident reports (0.84 vs. 1.15 per 1000 patient days) among infants in the FIC group from enrolment until discharge, but neither was statistically significant in this pilot study (p = 0.057 and p = 0.78, respectively).

### Maternal measures

#### Maternal demographics

Of the 42 mothers, all were married or in a common law relationship and 17 (40%) had at least 1 other child aged between 2 and 15 years. The families lived at variable distances from the hospital, from 15 minutes by subway to 2 hours by car. Only 22 of the mothers were Canadian born, although 11 had been resident in Canada for more than 10 years. The ethnic background of the mothers was varied, with 22 identifying themselves as Caucasian. All had at least a grade 10 high school education, with 6 having a graduate education, and 27 (71%) were employed outside the home at the time of birth. The mothers varied in age from 23 to 45 years old, with a mean (SD) age of 34 (5.3) at the time of enrolment.

#### Parent stress scores

Maternal stress scores measured on enrolment among the mothers in the FIC group (n = 42) were not significantly different compared with mothers in the control group (n = 14): 3.06 ± 0.12 vs. 3.25 ± 0.19, respectively, p > 0.05. However, by discharge the mean parental stress score had fallen to 2.30 ± 0.13 for the FIC mothers (p < 0.01, compared with enrolment). In contrast, no significant change was noted in the control group (3.25 ± 0.19 at admission, 2.99 ± 0.2 on discharge, p > 0.05).

### Program feasibility and evaluation

The data collected during semi-structured interviews with parents and nurses indicated that the FIC program was acceptable as a model of care to both groups. Specific parent and nurse feedback on the implementation of the program was incorporated into program improvements made during the pilot study. For example, the parent curriculum was updated and improvements were made to the parent room. A complete qualitative evaluation of the FIC educational program for parents and nurses and the parent-to-parent support provided by the veteran parents will be published elsewhere, but a brief overview is provided here.

From the parents’ responses to the open ended question “Describe your experience of FIC” several themes were identified, including the knowledge and confidence parents gained from the program, and the change in their relationship with the medical care team and other parents. A total of 35 of the 42 parents interviewed specifically reported that “gaining knowledge and confidence” was a big part of their experience. Twenty parents also indicated that being able to provide “hands-on” care was really important.

“I really enjoyed being part of the project, I feel like it's given me more confidence going home with a prem, or guess he’s not a prem any more. Yeh, I just feel a lot more confident handling him, a lot more confident in my own routines at the hospital and yeah, just a lot more confident going home”

*“Before when we were in NICU we were like all over the place, we didn’t know what to do, and the needles and IVs and everything that was a really painful procedure for my baby and for us to look at it. Once I joined the program then literally, it really helped me and made me really calm down, less stressful I am right now* [less] *than I was before”*

Another important theme that was identified by the parents was the change in their relationship with the medical care team and with other parents.

*“I’ve loved being in the* [FIC] *project, being able to do rounds is one thing that I have liked the most and having the doctors and residents actually really listening to what you have to say.”*

All of the 19 nurses who were interviewed described the benefits of the program both for themselves and families. Over 50% of the respondents commented on the closer connection that they felt to parents who were participating in the FIC program, and 13 out of the 19 nurses commented that their role had changed and that they were doing less hands-on care and more teaching.

“I was more connected and bonded more with the parents than I would have with the regular babies. They are there all the time and you rely on their information much more, mostly because of your bond and you know their knowledge and their confidence. It has made a huge difference with my relationship with the parents”

“I had to do less hands-on care for sure. My responsibilities making sure that meds are given and the babies are still fine, the mom is comfortable, that hasn’t changed at all. The overall perspective hasn’t changed but there is less hands on care”

## Discussion

Our aim in this study was to develop and pilot test FIC in a Canadian NICU. It is a model of care that addresses the need to facilitate a care partnership with parents of NICU infants and promote maternal development in the NICU [20,21]. Unlike other cultural settings where FIC has been adopted, our discussions with families and NICU staff indicated that to make such a model feasible in Canada, in addition to physical and environmental supports, we needed to add other dimensions to our program, specifically parent education, parent-to-parent support, and nursing education. We also had to adapt the requirements of the program to enable families to participate in their infant’s care despite not being able to commit to living at the hospital 24 hours a day, which is a requirement in other care-by-parent models [2,8]. However, this study demonstrates that with these modifications, the introduction of FIC to a Canadian NICU is feasible. In addition, our study suggests that FIC appears to improve the short-term outcomes for both infants and their parents in the NICU.

Previous reports in the literature on the topic of family-centred care interventions in the NICU appear to be grouped into 2 major fields, those focusing on the provision of parent education and those more focused on the care-by-parent model, whereas our FIC program combined the two. The literature on the benefits of providing educational interventions alone to parents of preterm infants is mixed. In a cluster randomised trial across 6 neonatal centres in the UK, of a parenting educational intervention to enhance parental care of the infant, Glazebrook *et al*,*.* were unable to demonstrate any significant changes in parent or infant outcomes [22]. A recent study of a skill-based program called “cues” was also unable to demonstrate any additional benefit over non-specific education [23]. However, in a randomised controlled trial, Melnyk *et al.*, demonstrated that the COPE program, which focuses on providing parents with a very structured educational-behavioural program, improves parent coping and mental health outcomes during and after hospitalization, as well as shortening infants’ length of stay [6]. Whether the use of a specific educational program alone without the additional expectations of parent involvement in their infant’s care will translate into similar benefits in other outcomes, as observed in our study, remains to be seen. What we have heard from our parents is that although the education sessions were useful, being able to participate as part of the care team and having clear expectations of their role was even more important.

The reports of care-by-parent models in the literature, such as those by Levin *et al.*, [2] and Ortenstrand *et al.*, [8], have not specifically described a program of parent education but do require parents to be present in the hospital 24 hours a day. A randomised controlled trial of the “Stockholm” model by Ortenstrand *et al*., reported a shorter length of stay in hospital and a decrease in bronchopulmonary dysplasia in infants cared for by their parents, suggesting that parental involvement can decrease some of the short and long-term morbidities of preterm birth. Our FIC program supports parents’ ability to spend more time at the bedside by providing clear expectations of the parent, particularly an expectation of a time commitment, as well as rest space and psychosocial supports, but we did not expect parents to provide 24-hour care. The decision to not require 24-hour care was based on many different factors; the physical limitations of our unit, the social context of our families living in a city often with limited extended family support (particularly as many of these families had other children); and the perceived cultural unacceptability of making such a demand of families. However, research staff observed that most of the parents gradually increased the amount of time they spent in hospital beyond the required 8 hours, particularly as their infants’ progress with oral feeding required their extended presence, although this was not formally assessed.

As this was a pilot study and the sample size was small, statistical power was limited; however, it is important to note the interaction of weight gain and time, indicating a greater improvement in the FIC group over time. A larger trial will clarify whether this is a true benefit of the FIC program. However, the significant increase in breast feeding rates is remarkable given how difficult it can be to establish breast feeding in preterm infants [24]. The provision of breast milk feeds to preterm infants has been shown to decrease morbidities including severe ROP and infection, and to improve their neurodevelopmental outcome [25-27]. Another important outcome of this study was the effect of the FIC program on parental stress. The significant decrease in stress scores of FIC parents over their time in the unit (vs. no change in controls) suggests a benefit of the FIC program, which according to the parents enabled them to have greater confidence in their parenting skills on discharge from the hospital. The parents’ reflections on their experience also indicate that the program appeared to achieve what it was intended to do, that is to enable parents to participate in their infants’ medical care while in the NICU. Our anticipation of the need for greater parental education to allow them to take on this role was also noted. The acceptability of the model to nursing staff was also key to its success. Despite the small sample, the nursing interviews indicated that implementation of the FIC program allowed the role of nurses to shift from caregiver to facilitator and coach for parental involvement.

The study results may have been confounded by unmeasured variables, such as other causes of prolonged hospitalisation in the control infants. This is particularly worth considering in regards to the effects of the intervention on severe ROP. While there is some justification for why an intervention such as the FIC program might affect neuronal developmental, it is also possible that the control infants were somehow different. In terms of stress reduction among FIC parents, the persistently high stress scores in the concurrent control parents could possibly be attributed to their infants being sicker, which was not measured. Another limitation of this study was the use of critical incidence reports as the only measure to monitor safety. However, while statistical power was limited and other safety indicators were not measured, the number of critical incident reports is the main indicator system used by hospitals to monitor safety and the study results suggest that, at the least, there was no increase in critical incidents during the implementation of FIC.

A final limitation of this study is that the enrolled parents, although of a very broad demographic profile, may not have been representative of our NICU parents in general. Although we had 75% enrolment of those families we approached, we did not approach every eligible family in the NICU as we had so few beds available for the program. Thus, we cannot conclude that the results of this study are generalizable to all parents in all NICU settings. However the results are consistent with the literature, which indicates that modifications of the NICU environment and greater parental holding, attachment, and responsiveness can improve infant outcomes [28-31]. Feedback from the participants also indicates that shifting the role of parents from passive support to active caregiving is feasible in the Canadian setting; consequently, the FIC model merits further study.

## Conclusions

This study suggests that FIC is feasible and safe in Canada and results in improved weight gain among preterm infants. This innovation has the potential to improve not only weight gain and breast feeding, but also other short and long-term infant and family outcomes. A multi-centre randomized controlled trial is needed to further evaluate the efficacy of FIC.

## List of abbreviations

BPD: bronchopulmonary dysplasia; CI: confidence interval; COPE: Creating Opportunities for Personal Empowerment program; CPAP: continuous positive airway pressure; FIC: family integrated care; IQ: interquartile range; IVH: intraventricular hemorrhage; N/A: not applicable; NEC: necrotizing enterocolitis; NICU: neonatal intensive care unit; PSS-NICU: Parental Stress Scale: Neonatal Intensive Care Unit; PVL: periventricular leukomalacia; ROP(3): retinopathy of prematurity (stage 3); SD: standard deviation; SNAPII: score of neonatal acute physiology version II; Wt1: weight on enrolment; Wt21: weight 21 days after enrolment; Zwt1: z-score for weight on enrolment; Zwt21: z-score for weight 21 days after enrolment.

## Competing interests

The authors declare that they have no competing interests.

## Authors’ contributions

KOB participated in the study design, coordination, and analysis of the data, and drafted the manuscript. MB participated in the study design and oversight. KM participated in the study design and oversight. TM participated in the study design and oversight. LOL participated in the study design and oversight. KC participated in the study design and oversight. MG participated in the study design and oversight. TD participated in the study design and oversight. AL guided the development of the research proposal. SKL is the principal investigator of the trial, conceived of the research proposal and study design, obtained the research grant, analysed the data, and participated in preparation of the manuscript. All authors have read and approved the final manuscript.

## Declarations

This article has been published as part of *BMC Pregnancy and Childbirth* Volume 13 Supplement 1, 2013: Preterm Birth: Interdisciplinary Research from the Preterm Birth and Healthy Outcomes Team (PreHOT). The full contents of the supplement are available online at http://www.biomedcentral.com/bmcpregnancychildbirth/supplements/13/S1.

All of the publication fees will be funded by the Preterm Birth and Healthy Outcomes Team Interdisciplinary Team Grant (#200700595) from Alberta Innovates - Health Solutions, formerly the Alberta Heritage Foundation for Medical Research.
